# Recent Findings in the Genetics of Blood Pressure: How to Apply in Practice or Is a Moonshot Required?

**DOI:** 10.1007/s11906-018-0863-1

**Published:** 2018-06-07

**Authors:** Sandosh Padmanabhan, Alisha Aman, Anna F. Dominiczak

**Affiliations:** 1 Institute of Cardiovascular and Medical Sciences, University of Glasgow, 126 University Place, Glasgow, G12 8TA UK; 2 University of Glasgow, Wolfson Medical School Building, University Avenue, Glasgow, G12 8QQ UK

**Keywords:** Hypertension, Blood pressure, Genetics, Genetic risk score, Global disease burden, Hypertension moonshot

## Abstract

**Purpose of Review:**

Hypertension is recognised as the biggest contributor to the global burden of disease, but it is controlled in less than a fifth of patients worldwide, despite being relatively easy to detect and the availability of inexpensive safe generic drugs. Blood pressure is regulated by a complex network of physiologic pathways with currently available drugs targeting key receptors or enzymes in the top pathways. Major advances in the dissection of both monogenic and polygenic determinants of blood pressure regulation and variation have not resulted in rapid translation of these discoveries into clinical applications or precision medicine.

**Recent Findings:**

Uromodulin is an example of a novel gene for hypertension identified from genome-wide association studies, currently the basis of a clinical trial to reposition loop diuretics in hypertension management. Gene-editing studies have established a genome-wide association studies (GWAS) SNP in chromosome 6p24, implicated in six conditions including hypertension, as a distal regulator of the endothelin-1 gene around 3000 base pairs away. Genomics of aldosterone-producing adenomas bring to focus the paradox in genomic medicine where availability of cheap generic drugs may render precision medicine uneconomical.

**Summary:**

The speed of technology-driven genomic discoveries and the sluggish traditional pathways of drug development and translation need harmonisation to make a timely and early impact on global public health. This requires a directed collaborative effort for which we propose a hypertension moonshot to make a quantum leap in hypertension management and cardiovascular risk reduction by bringing together traditional bioscience, omics, engineering, digital technology and data science.

## Introduction

Blood pressure (BP) is regulated by a complex network of interacting pathways involving renal, neural, endocrine, vascular and other mechanisms along with multiple environmental factors. Multiple genes within each of these systems contribute to BP regulation and the identification of these variants, their effects and potential targets for drugs has been the goal of genetic studies of BP and hypertension. Hypertension is the single largest cause of mortality worldwide and the public health challenges in reducing the burden of hypertension require new discoveries for both detection and treatment. Genomic studies have taken precedence over other discovery sciences in trying to address these challenges. The key reasons are that the genome essentially remains unchanged throughout life, genomic variations that influence gene function and thence phenotype have the potential to be targeted by drugs and finally, the rapid revolution in high-throughput genomics in the last 20 years. In this review, we assess the role of genomics of hypertension in the context of current discoveries and focus on four exemplars where whole genome studies are advancing our knowledge of BP regulation with potential early clinical applications. We finally discuss what is required to move discovery to clinical implementation with demonstrable early and tangible impact on public health.

## SNPs—‘Drinking From the Fire Hose’

**Fig. 1:**
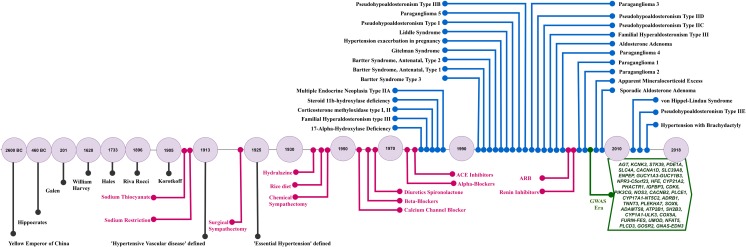
Hypertension clinical, therapeutic and genomic timeline. Current hypertension pharmacotherapy whilst not informed by genomics does target many of the pathways involved in monogenic BP syndromes. The timeline highlights the potential of the modern genomics era for discovery of novel pathways and new drugs for hypertension

The era of genome-wide association studies (GWAS) commenced in 2007, resulting in a torrent of GWAS for a wide range of polygenic traits including hypertension and BP. The hypothesis underlying GWAS is that common variations (SNPs) can have significant impact on common traits and thence human health. Currently, there are over 500 SNPs associated with BP and hypertension [1••] with more likely to be identified when the full value of the UK Bio-Bank genetic data is harnessed. The scale and rate of SNP associations for BP (and for other similar polygenic traits) have been likened to ‘drinking from the fire hose’ [2••]. Soon, we expect an acceleration in identification of even more genomic signals from the combination of whole-genome and omics studies on millions of individuals. The ‘drinking from the fire hose’ analogy is as relevant today as it was in 2007 and highlights the reality of modern genomic studies where physicians and scientists try to keep up with exponential growth in genetic discoveries. The ultimate goal of any genetic study is clinical translation and application. However, technically, GWAS is not designed to inform clinical practice directly in the doctor’s office. It is designed to explore genetic architecture at a population level and not at an individual level. Furthermore, the results of GWAS studies do not directly yield a causal gene target or mechanism. This results in an inevitable time lag between GWAS discoveries progressing through underpinning biological studies followed by clinical translation. Most common traits including hypertension demonstrate a handful of early promising functional signals which are the ‘low hanging fruit’ of potentially translatable signals amongst the hundreds of GWAS signals. In a recent review, overlaying the clinical, therapeutic and molecular genetic timelines of hypertension understanding and scientific progress highlighted three points—(i) hypertension and its complications has been recognised over 2000 years ago; (ii) despite major advances in unravelling the molecular determinants of monogenic forms of hypertension, these have not informed new drug discoveries, and (iii) anti-hypertensive drugs used currently target most of the pathways involved in monogenic hypertension and there have been no new molecular targets for hypertension identified in the last decade [3]. The explosion of genetic discoveries from genomic studies (GWAS, exome and whole genome studies) is likely to catalyse the next era in BP prevention, detection and management. The biggest bottleneck so far has been linking GWAS SNPs to biology, and this is slow despite recent efforts such as ENCODE [4], Epigenome Road Map [5], and GTEx [6] allowing functional exploration of risk variants at genome-wide scale. Though most genetic studies, so far, have focussed on single variant associations, and not on epistasis or gene-environment interactions, there are now efforts to extend studies on these domains. Such studies may identify more new signals, but more importantly bring the environment aspect into consideration (Fig. 1).

## GWAS SNP to Clinical Trial

A GWAS for hypertension using an extreme case-control design identified a SNP in the 5′ region of uromodulin gene (*UMOD*), which is almost exclusively expressed in the thick ascending limb of the loop of Henle in the kidney. This study identified a potentially novel pathway of BP regulation, which acts through an effect on sodium homeostasis [7]. Moreover, independent studies have identified SNPs highly correlated with the hypertension SNP near *UMOD* to be associated with chronic kidney disease [8]. Trudu et al. [9] showed that furosemide treatment significantly enhanced natriuresis and reduced BP levels both in the transgenic mice and in the hypertensive individuals homozygous for the *UMOD* increasing allele, making this a potentially interesting locus for both hypertension and renal disease [7–9]. This is now the basis of a clinical trial (www.clinicaltrials.govNCT03354897) to reposition a loop diuretic in the hypertension care pathway.

## Novel Pathways with Potential Translation

The vascular endothelium synthesises and releases a spectrum of vasoactive substances, including nitric oxide (NO), a potent vasodilator. Other vascular relaxation factors include endothelins and prostacyclin. Endothelin-1 (*EDN1*) activates specific ETA receptors (*EDNRA*) on vascular smooth muscle cells to cause vasoconstriction and cell proliferation. In contrast, endothelial ETB receptors (*EDNRB*) mediate vasodilatation via release of NO and prostacyclin (*PGI2*). A GWAS of coronary artery disease (CAD) showed the SNP rs9349379 G allele (frequency 36%) was associated with increased risk of CAD and coronary calcification but decreased risk for four conditions (migraine headache, cervical artery dissection, fibromuscular dysplasia and hypertension [10, 11]). This SNP is located within the third intron of the gene encoding phosphatase and actin regulatory protein 1 (*PHACTR1*). However, functional analysis of this variant showed that it regulates expression of endothelin 1 (*EDN1*), a gene located 600 kb upstream of *PHACTR1*. This result was obtained using the new CRISPR/Cas9 gene-editing method to delete a small region of DNA at rs9349379 in human pluripotent stem cells, which were then converted into vascular cell to study how rs9349379 regulates the activity of the physically distant *EDN1* gene. Interestingly, variants in the *PHACTR1* gene have been associated with fibromuscular dysplasia (FMD), a non-atherosclerotic vascular disease leading to stenosis, dissections and aneurysms affecting mainly the renal and cerebrovascular arteries [12]. This study highlights the complexity of functional analysis of GWAS signals and the value of establishing the true causal gene linked to the GWAS signal. Endothelin receptor antagonists were discovered in the 1980s and are now used in the treatment of pulmonary hypertension. The early trials for hypertension treatment were not encouraging, but it is clear that greater understanding of endothelin regulation from genomic studies may inform future targeted use of endothelin antagonists in the treatment of hypertension.

## Aldosteronism

Aldosterone-producing adenomas (APAs) are found in 1.5–3.0% of hypertensive patients in primary care and can be cured by surgery. Elucidation of genetic events may improve our understanding of these tumours and ultimately improve patient care. A gain-of-function somatic mutation in a K^+^ channel, *KCNJ5*, which results in membrane depolarization and enhanced aldosterone production, is a common genetic defect noted for ~ 40% of APAs. Mutations in three other genes, encoding the α-subunit of Na^+^-K^+^-ATPase itself; the *ATP2B3*, a plasma membrane Ca^2+^-ATPase homologous to the sarcoplasmic endoplasmic reticulum Ca^2+^-ATPases (SERCA); and *CACNA1D*, encoding an L-type Ca^2+^ channel CaV1.3, are observed in ~ 7% of the cases [13]. Whereas APAs in adrenal zona glomerulosa cells harbour gain-of-function mutations in genes important for the regulation of Na^+^ and Ca^2+^-, *ATP1A1* and *CACNA1D*, respectively, *KCNJ5* mutations are common in APAs resembling cortisol-secreting cells of the adrenal zona fasciculata [14••]. Adenomas with *KCNJ5* mutations are typical of Conn’s adenoma and tend to be larger than other tumours with fasciculata-like features by histopathology and gene expression analysis, whilst *CACNA1D* and *ATP1A1* mutations appear to be associated with a glomerulosa-like phenotype and are more likely to be diagnosed in older men with resistant hypertension [15, 16]. The case of APAs brings to focus a paradox in genomics medicine, the availability of a specific and cheap pharmacologic antagonist of aldosterone, spironolactone, and benign nature of APAs, create a hurdle to embarking on the expensive investigations and surgery, which at best offers 30 to 60% likelihood of curing hypertension [14••].

## Mood Disorders and Hypertension Genetics

Although a large body of work has identified associations between mood disorders and cardio-metabolic diseases (such as coronary heart disease and diabetes), the relationship between major depressive disorder/bipolar disorder (MDD/BD) and hypertension has been much less studied. There is however now good evidence of pathophysiological overlap between MDD/BD and hypertension. In clinical practice, changes in mood (both depressive and manic) have long been recognised as side effects of some anti-hypertensive medications [17] and recent epidemiological work has identified that hypertension is a common comorbidity for individuals with MDD and BD [18]. Furthermore, recent GWASs have established that a locus containing the gene *CACNA1C* (coding for a subunit of the L-type calcium channel) is a risk factor for BD [19, 20]. Similarly, *CACNB2*, also a voltage-gated calcium channel, is a risk factor for both mood disorders and hypertension. The pathophysiology of hypertension has therefore emerged as an important new potential avenue for developing novel treatment approaches for mood disorders. For example, a recent pilot investigation of the L-type calcium-channel blocker Isradipine suggested that it may be effective for bipolar depression [21]. There is also now good evidence from animal models that drugs acting on the renin-angiotensin system (such as ACE-inhibitors) have antidepressant properties [22]. Using data on 144,066 patients from the Glasgow Blood Pressure Clinic Cohort, we recently identified that treatment with ACE inhibitors or angiotensin receptor blockers (ARB) (compared to other classes of anti-hypertensives) is associated with significantly lower rates of new-onset hospital admission for mood disorders [23].

## Genetic Risk Prediction

Summarising the plethora of genetic findings into a single metric is attractive as it potentially produces an individual risk score that could inform personalised treatment or prevention. A polygenic genotype risk score (GRS) is calculated from GWAS summary statistics by summing the number of risk alleles carried by an individual, weighted by the GWAS effect size. The GRS, by summarising several million genotyped and imputed common genetic variants into a single variable for an individual, is very attractive because of the potential for risk prediction and individualised preventive measures. By rescaling GRS to have a mean of 0 and a standard deviation of 1, an individual’s GRS can be converted to quantiles that enable separation of groups with the highest and lowest risks. The GRS of the majority of individuals will be close to the population mean, implying that their predicted risk will be close to the population lifetime disease risk. It is also pertinent to note that the relative risk estimates of GWAS SNPs and GRS are generally < 2 which signifies trivial clinical utility of these with no real relevance to either screening and diagnostic tests, and hence, the realistic conclusion that GRS is not particularly informative for an individual.

A possible clinical use of the GRS is in determining where an individual’s GRS lies on the population distribution. For example, preventive measures or regular screening or early treatment may be offered to those individuals in the top 1 or 5% of GRS values, depending on the estimated risk for the disease and its severity. Two studies, one using CAD GRS constructed from 50 CAD risk variants [24••] and another using a GRS constructed from 314 BP risk alleles [25••] showed that lifestyle factors made a similar contribution irrespective of the patient’s genetic profile to CAD risk or BP respectively. They suggest that lifestyle measures offer the same benefit from prevention across genetic risk strata. However, this interpretation need to be contextualised on three key issues about lifestyle and prevention—the difficulty in accurately measuring lifestyle exposure, the extrapolation of a single time point measure to lifetime exposure and the fact that lowering BP by lifestyle requires an enormous sustained voluntary effort by the individual. Nevertheless, these studies exemplify the progress made in genomics and these small incremental steps may be the harbinger of major future advances.

## The Case for a Hypertension Moonshot

Hypertension is a study in contrasts. It is the leading single risk factor for mortality and global burden of disease with over 1 billion people affected worldwide. The diagnosis of hypertension is still based on measurement using a cuff invented in 1896. The revolution in genomics has resulted in over 500 SNPs associated with BP and over 32 genes associated with monogenic forms of hypertension which is still awaiting entry into clinical applications. Hypertension is one of the diseases whose treatment has been tested by the largest number of randomised clinical trials. However, BP-lowering drugs that are effective and inexpensive are offered to, and regularly used by, only a fraction of the patients who need them. Pharmaceutical companies have largely abandoned the search for a “blockbuster” drug. New drugs and device therapies in development whilst initially promising have not been able to survive the rigour of sham-controlled trials so far. Because of costs, even if successful, they are likely to be directed towards a relatively small minority of the hypertensive population. The differential in the apparent plodding rate of progress in hypertension detection and management and the whirlwind of exploratory, discovery studies from genomics and big data needs to be reconciled for any meaningful leap in hypertension management and cardiovascular risk reduction. This necessitates the pace of progress in disparate but essential strands of basic science, digital technology, innovation, evidence generation and synthesis, clinical, public health and health economic activities to keep pace with each other. A recognition of the scale of effort required to solve the hypertension problem is reflected in the International Society of Hypertension’s global synchronised screening campaign which took place for the first time during May 2017 in over 100 countries and screened over 1.2 million people (http://maymeasure.com/about/). Even though this is only a screening programme with plans to continue annually, identifying a large proportion of the 1.2 billion hypertensives worldwide will have implications on the healthcare systems. Solutions are needed on a number of strands to ensure that all individuals who become aware of their BP know what action to take and that this does not unduly burden overstretched healthcare systems. The challenge is to develop and implement these solutions on a global scale quickly. This means making a decade worth of discoveries, validation, testing and implementation in 5 years or less which requires a coming together of the empirical, all-comers, RCT-informed strategies and individualised, multi-omics, big data and technology-driven sciences. Realisation of this goal requires transformative advances focussed on collecting more patient data and enabling faster research, better decision support for physicians and better engagement of patients on a global scale. Thus, there is a strong case for a ‘hypertension moonshot’ (along the lines of the cancer moonshot) to accelerate screening, diagnosis and management of hypertension. Some of the challenges for the moonshot include newer ways of measuring BP, use of multidimensional data from wearables in phenotyping and monitoring, refining the taxonomy of hypertension using omics and big data, functional dissection and translation of genomic discoveries, and innovative clinical trials to accelerate clinical implementation from prediction to treatment. This would require investigators and stakeholders in biomedical, omics, healthcare, public health, regulatory, digital technology, engineering and pharmaceutical domains to work together in an ecosystem along with patients to identify and overcome bottlenecks. The gains from this will be immediate rather than in the distant future, and developing the multi-disciplinary teams necessary to deliver this over the next 5 years will be an ambitious but not insurmountable challenge.

## Conclusions

As hypertension shows no geographic boundaries, the solution to mitigate its risk should be global in scope. Advances in high-throughput genomics have vastly increased our understanding of the genetic architecture of BP and hypertension. The aspiration that this would tangibly improve hypertension management and thence its public health impact has not yet been realised. The burgeoning list of genomic variants associated with BP and hypertension provides a realistic basis for refining the molecular taxonomy of hypertension. However, realistically, any new taxonomy of hypertension should also incorporate patient characteristics including social, lifestyle and environmental influences in addition to molecular and genomic information. Given the global burden of hypertension, a moonshot initiative is essential to harness expertise and knowledge in diverse strands to bring about a rapid and positive impact on global cardiovascular health.
